# Identification of Bioactive Compounds of *Asparagus officinalis* L.: Permutation Test Allows Differentiation among “Triguero” and Hybrid Green Varieties

**DOI:** 10.3390/molecules26061640

**Published:** 2021-03-15

**Authors:** Cecilia Jiménez-Sánchez, Fabián Pedregosa, Isabel Borrás-Linares, Jesús Lozano-Sánchez, Antonio Segura-Carretero

**Affiliations:** 1 Department of Analytical Chemistry, Granada University, Avda Fuentenueva s/n, 18071 Granada, Spain; cecilia.jimenez-sanchez@unige.ch; 2 Functional Food and Development centre (CIDAF), Health Science Technological Park, Avda del Conocimiento s/n, 18016 Granada, Spain; iborras@ugr.es; 3 EECS Department, University of California, Berkeley, CA 94720-1776, USA; pedregosa@google.com

**Keywords:** asparagus, Asparagaceae, HPLC, mass spectrometry, classification

## Abstract

In this study, we determined the phytochemical profile of the Spanish “triguero” asparagus landrace “verde-morado” (*Asparagus officinalis* L.), a wild traditional landrace, and the improved “triguero” HT-801, together with two commercial green asparagus varieties. For comparison, we used reverse-phase high-performance liquid chromatography coupled with diode array electrospray time-of-flight mass spectrometry (RP-HPLC-DAD-ESI-TOF/MS) followed by a permutation test applied using a resampling methodology valid under a relaxed set of assumptions, such as i.i.d. errors (not necessarily normal) that are exchangeable under the null hypothesis. As a result, we postulate that “triguero” varieties (the improved HT-801 followed by its parent “verde-morado”) have a significantly different phytochemical profile from that of the other two commercial hybrid green varieties. In particular, we found compounds specific to the “triguero” varieties, such as feruloylhexosylhexose isomers, or isorhamnetin-3-*O*-glucoside, which was found only in the “triguero” variety HT-801. Although studies relating the phytochemical content of “triguero” asparagus varieties to its health-promoting effects are required, this characteristic phytochemical profile can be used for differentiating and revalorizating these asparagus cultivars.

## 1. Introduction

Cultivated “triguero” asparagus comes from the wild autochthonous asparagus (*Asparagus officinalis* L.) from Huétor-Tajar in Andalusia, Spain. While some research suggests that “triguero” asparagus could be a hybrid between cultivated diploid varieties of *Asparagus officinalis* *L*. and the wild species *Asparagus maritimus* [1], others have not confirmed this fact [2]. This traditionally grown, widely consumed variety has a slightly bitter taste, is medium to small in diameter, and ranges from light green to purple in color; hence, it is also known as “verde-morado” (purplish-green). Since 1999, botanists have been working on the genetic improvement of this “verde-morado” landrace by crossbreeding, the first result being the new variety HT-801. This new variety purportedly has higher concentrations of bioactive compounds while maintaining the organoleptic characteristics of its parent landrace “verde-morado”. Previous research from our research group has tentatively identified a total of 94 compounds belonging to different chemical classes, such as organic acids, amino acids, peptides and derivatives, polyphenols (hydroxycinnamic acids, flavonols, lignans, and norlignans), oxylipins, and others, using a reverse-phase high-performance liquid chromatography coupled with electrospray quadrupole time-of-flight mass spectrometry (RP-HPLC-ESI-QTOF/MS2) methodology of commercial green asparagus (*Asparagus officinalis* L.) [3]. Although some research has claimed that some “triguero” asparagus contains certain bioactive constituents, such as dietary fiber, saponins, sterols, oligosaccharides, carotenoids, amino acids, and polyphenols, including mono-, di-, and triglycosides of quercetin, isorhamnetin, and kaempferol [4,5,6], a full characterization of this landrace has not yet been completed. The chemical characterization and the search for bioactive components specific to these native asparagus may enable new criteria to be established for selection and help promote the cultivation and consumption of a high-quality product that is still poorly known, and whose cultivation is challenging and being replaced by commercial hybrids with more homogeneous yield and spears of greater caliber [5].

Comparisons of the bioactive compounds contained in different plant varieties require statistical analyses, e.g., one-way analysis of variance (ANOVA). However, the parametric ANOVA test is subjected to certain assumptions, i.e., approximately normal distribution of the dependent variable for each category, or homogeneity of variances. Permutation tests offer an alternative to parametric approaches when sample sizes are low and error terms do not fulfil distributional assumptions of the parametric approach [7]. These are computational resampling-based tests, which permute data falling under the null hypothesis of equal data distributions between groups [8], overcoming the limitations of parametric methods, in a simple and comprehensive way.

In this context, the aim of this study was to determine the phytochemical profile of the “triguero” asparagus landrace “verde-morado” and the new variety HT-801 together with one of two commercial green asparagus varieties, in order to make comparisons using an HPLC–ESI–DAD–TOF/MS approach. The permutation test was applied to establish significant differences between these asparagus and identify the phytochemicals responsible for these differences.

## 2. Results and Discussion

### 2.1. Analytical Characterization

**Figure 1:**
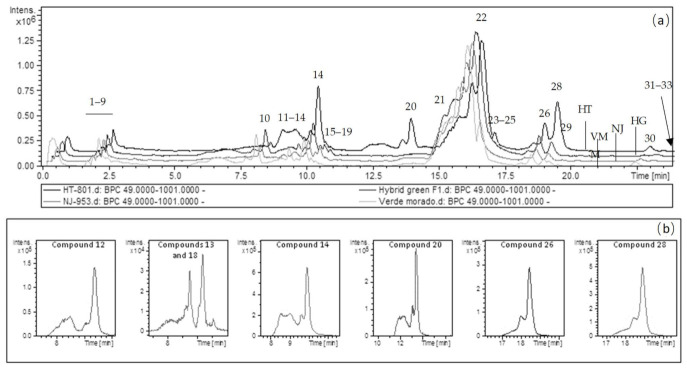
(**a**) HPLC-ESI-TOF/MS base peak chromatogram (BPC) in negative ion polarity of the four varieties studied: HT–801 (HT), Hybrid green (HG), NJ–953 (NJ), and Verde-morado (VM). (**b**) Extracted ion chromatogram (EIC) of selected compounds in the representative variety HT–801 (HT). From left to right: compound **12** (*p*-coumaroylquinic acid), compounds **13** and **18** (feruloylhexosylhexose isomers 1 and 2), compound **14** (feruloylquinic acid), compound **20** (quercetin rhamnosyl rutinoside isomer 1), compound **26** (kaempferol-3-*O*-rutinoside [nicotiflorin]), and compound **28** (isorhamnetin-3-*O*-rutinoside [narcissin]). Peak number, retention time, *m/z*, molecular formula, and absorbance of each peak are provided in Table S1. Abbreviations: HT, HT-801; HG, hybrid green F1; NJ, NJ-953; VM, “verde morado”.

Asparagus extracts were analyzed using an HPLC system coupled to DAD and ESI-TOF-MS analyzers (HPLC–DAD–ESI-TOF-MS) in the negative ionization mode in order to identify the polar fraction. Peak identification was performed on the basis of their relative retention time values, and their UV–vis spectra and mass spectra obtained using TOF-MS together with information previously reported in the literature (Table S1 and Figure 1). In agreement with the literature [9,10], a certain tendency was observed in the elution order of the compounds related to their chemical structure class, appearing in the following order of increasing retention time (RT), and thus hydrophobicity: amino acids, sugars, organic acids, phenolic acid derivatives except for glycerol derivatives, flavonoids, and phenolic acid glycerol esters (Table S1). The time-of-flight (TOF) mass analyzer provides excellent mass accuracy over a wide dynamic range, and measurements of the true isotope pattern elucidate the molecular formulas of unknown metabolites with a high degree of reliability. The DAD allowed us to record the UV/Vis spectrum of every peak in the chromatogram in the range of 190–950 nm and thus attribute each to a certain class of compound, since every type of chromophore exhibits a characteristic spectrum and absorbance maximum. Nevertheless, as peak resolution was not complete in some cases and thus the UV/Vis spectrum of coeluting compounds might hide the spectrum of other analytes, the absorbance maximum was not established for all compounds. 

#### 2.1.1. Amino Acids

Free amino acids are commonly found in plants and are reportedly indicative of fruit growth, maturation, and ripening [11]. Peaks 1, 2, and 4 in the chromatographic run have been proposed as l-arginine, l-asparagine, and l-glutamic acid. They showed deprotonated molecules at *m/z* 135, 141, and 146, respectively. Amino acids have been previously detected at 210–220 nm [12], in agreement with our results, in which a band of between 210 and 220 nm appeared in the four peaks. These molecules are among the most polar naturally occurring amino acids according to Venn’s diagram, which represents the different relationships among the amino acids [13]. It seems natural that the polar extraction was successful with the most polar amino acids that were then detected. Results reported by some researchers [14,15] show that green asparagus has a high protein content, ranging from 428.80 to 509.35 mg/g dry weight. 

#### 2.1.2. Carbohydrates

Free low-molecular-weight carbohydrates are biologically important constituents of vegetables. Minor compounds in this fraction are oligosaccharides, as well as cyclitols, alditols, and acid sugars, many of these having positive properties [16]. In our research, saccharides, acid sugars, and other minor carbohydrates have been found in asparagus samples. Carbohydrates preceded polyphenols in the chromatographic run, due to their highly hydrophilic character. Peaks 3, 6, 7, and 8 were tentatively characterized as carbohydrates. More concretely, compounds **3** and **6** showed *m/z* at 135 and 165, respectively, and were identified as acid sugars, specifically aldonic acids where the aldehyde of the aldose is oxidized to form a carboxylic acid functional group (threonic and xylonic/arabonic/ribonic acid, respectively). These molecules have been found to be part of the plant’s metabolome [17,18]. A common soluble saccharide found in our asparagus samples was also found at m/z 341 and was characterized as a disaccharide (peak 7). Researchers have previously confirmed the presence of common soluble sugars in asparagus samples [19]. This might be explained by the fact that the hydrolysis of the polysaccharidic cell-wall material occurred as a result of metabolizing enzyme activities and postharvest treatments [20]. A carbohydrate with a deprotonated molecule at *m/z* 209, compatible with a heptulose (sedoheptulose), was also detected. Although this molecule has not been described in asparagus samples, it has been quantified in other vegetable samples belonging to the same genetic filo (cabbage, radish, spinach, lettuce, and chicory) [16].

#### 2.1.3. Organic Acids

Organic acids are known for their influence on the organoleptic properties of edible plants, being responsible for sourness or acidity [9]. Various organic acids and derivatives were found, more specifically quinic, malic, and abscisic acids, corresponding to peaks 5, 9, and 29, respectively, ranked by elution order. They yielded deprotonated molecules at *m/z* 191, 133, and 263. Quinic and malic acids eluted from the chromatographic column within the first 2.5 min of the run due to their highly polar character, while abscisic acid eluted at 19.4 min owing to a higher degree of hydrophobicity provided by the four methyl groups and their complex backbone structure. While the absorbance of quinic and malic acids was not detected, abscisic acid presented an absorbance maxima peak at 262 nm, in agreement with the literature [20]. Abscisic acid is a well-documented phytohormone, which has previously been reported in asparagus spears [21], although no prior evidence of the presence of quinic and malic acids is available in the literature.

#### 2.1.4. Phenolic Compounds

In this study, phenolic acid derivatives and flavonoids were detected. These molecules play an important role in antioxidation defense systems in plants. 

Regarding phenolic acid derivatives, all belonged to the hydroxycinnamic acid subclass. It has been reported that substantial amounts of hydroxycinnamic acids are intimately associated with plant-cell walls and can have a significant impact on their mechanical properties [22]. These compounds consisted of different hexose, quinic, tartaric, and glycerol derivatives of caffeic, ferulic, and coumaric acids. As it might be expected, hexose (peaks 13, 16, and 18), quinic (peaks 10, 12, and 14), and tartaric derivatives (peaks 11, 15, and 17) eluted much earlier from the column than glycerol derivatives, which eluted at the end (peaks 31–33). All derivatives showed the typical band I of absorption at 310–325 nm due to their cinnamoyl system, confirming their assignation as hydroxycinnamic acid derivatives, in agreement with previous reports [23]. In addition, derivatives of caffeic and ferulic acids showed a shoulder at 296 nm, typical of these compounds [24]. They were tentatively assigned as caffeoylquinic acid (chlorogenic acid) (peak 10), caffeoyltartaric acid isomers 1 and 2 (peaks 11, and 15), dicaffeoyltartaric acid (peak 17), feruloylhexosylhexose isomer 1 (peak 13), feruloylquinic acid (peak 14), feruloylhexose (peak 16), and feruloylhexosylhexose isomer 2 (peak 18). Furthermore, the one coumaric acid derivative did not show the shoulder at 296 nm, but it presented an absorbance maxima peak near 235 nm, consistent with the literature for this compound [25]. Among these compounds, there is previous evidence only for ferulic acid and its dimers in asparagus spears, which have been reported to be involved in postharvest textural changes through the formation of polysaccharide–lignin cross-links [26]. Finally, three chromatographic peaks corresponded to one or two molecules of ferulic and/or coumaric acid esterified to a glycerol backbone, namely dicoumarroyl glycerol (peak 31), coumaroylferuloyl glycerol (peak 32), and diferuloyl glycerol (peak 33). They showed the aforementioned bands at 310–320 nm and 296 nm, as well as an extra band that was not presented by the rest of the phenolic acid derivatives near 250 nm and that could be attributed to the glycerol residue. These unconventional phenolic acid derivatives have previously been isolated from aboveground parts of green asparagus [27]. 

All the flavonoids detected belonged to the flavonol subclass, which share the common skeleton of the flavonol nucleus but vary in the number and structure of their hydroxyl and methoxyl moieties. These were all quercetin, isorhamnetin, and kaempferol derivatives with different sugar substituents. As expected in reverse-phase liquid chromatography, glucosyl derivatives eluted earlier than rutinosyl and rhamnosyl derivatives, because of the larger numbers of hydroxyl groups. In addition, the elution behavior agreed with the polarity of the flavonol moieties, with quercetin derivatives eluting first, followed by kaempferol, and isorhamnetin derivatives. Flavonols present a band I at 347–370 nm and a band II at 250–267 nm [23]. For those with a substituted 3-hydroxyl group (glycosylated), the intensity of band I is somewhat lower than that of band II, which would easily characterize them as flavonol-3-*O*-glucosides. This type of glycosylation was attributed to some of these molecules. Therefore, flavonols were tentatively characterized as quercetin diglucoside (peak 19), quercetin rhamnosyl rutinoside isomer 1 (peak 20), quercetin glucosyl rutinoside (peak 21), quercetin-3-*O*-rutinoside (rutin) (peak 22), quercetin-3-*O*-glucoside (peak 23), quercetin rhamnosyl rutinoside isomer 2 (pek 24), isorhamnetin glucosyl rutinoside (peak 25), kaempferol-3-*O*-rutinoside (nicotiflorin) (peak 26), isorhamnetin-3-*O*-glucoside (peak 27), and isorhamnetin-3-*O*-rutinoside (narcissin) (peak 28). A previous report has provided evidence for some of these flavonols in asparagus samples [4]. It has been specifically reported that quercetin-3-*O*-rutinoside (rutin) represents 60–80% of the total phenolic content of asparagus extracts [4]. 

### 2.2. Permutation Test

It is well established that the phytochemical profile of a vegetable is influenced by genetic and environmental factors. Given that our samples were collected the same day in the same plot of land, and therefore grown under the same agricultural and environmental conditions, our results highlight the effect that genotype exerts on the phytochemical composition. To characterize the differences between varieties more accurately, we used permutation tests using the data of the concentration of the different bioactive compounds expressed as the peak area. This approach has been previously used in scientific research and it is indeed preferred to classical t or F tests from a statistical point of view [28,29]. To establish statistically significant differences in the compounds between the four varieties studied, we would normally use a one-way analysis of variance (ANOVA). In this test, the null hypothesis is that the means of the different compounds are identical, and the alternative hypothesis is that not all the means are identical. To determine a significance value for this test, the value of the F-statistic is compared against a null distribution that is known under the null hypothesis. However, in the case of the ANOVA, the null distribution is known to be subject to certain assumptions, such as normality of the residuals. Since these assumptions could not be verified, we opted instead to estimate the null distribution from the observed data using resampling methods. In particular, we used the permutation scheme for ANOVA detailed elsewhere [30]. Compared to a classical ANOVA, this method is valid under a relaxed set of assumptions, such as i.i.d. errors (not necessarily normal) that are exchangeable under the null hypothesis. As in the classical ANOVA, the test statistic used was the F-statistic, defined as the ratio between the within-group variability and the between-group variability. To compute the *p*-value associated with our hypothesis, we compared the value of F calculated on the original data with the distribution of values of F determined by permuting the observations that we denote Fπ. In other words, the distribution of Fπ under the null hypothesis was found by calculating all possible values of F under rearrangements of the labels on the observed data points. The probability associated with the null hypothesis is calculated as the proportion of the Fπ that are is than or equal to F, i.e.:

*p* = (no. of Fπ ≥ F) / (total no. of Fπ) (1)

In practice, not all possible permutations need to be taken, and typical choices are around 5000 permutations for tests with a level of significance ≤ 0.01 [31]. In our case, we took 100,000 permutations. Because the distribution of the test statistic was computed from the data itself and not assumed, the distributional assumptions of this test are much weaker than those for a classical ANOVA. In fact, the only assumption of the permutation test is that the observations are exchangeable under a true null hypothesis, rendering this technique highly robust.

**Figure 2:**
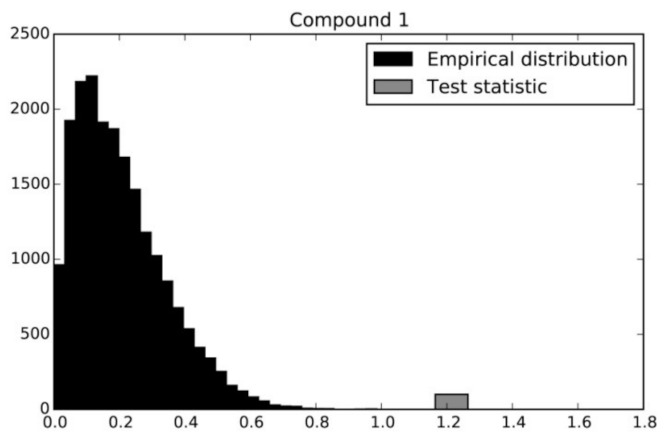
Permutation testing example for compound **1**. The empirical distribution of the F-statistic established by permutations for this compound can be seen in black. The value of the observed statistic is shown in gray.

This process is illustrated in Figure 2, where the empirical distribution of Fπ under the null hypothesis established by permutations for the first compound can be seen in black. The value of the observed F statistic is shown in gray. In this case, the associated *p*-value is < 10^−6^. This value means that the probability associated with the null hypothesis happens 1 time in 1,000,000 trials, and therefore the null hypothesis strains credibility and can be safely rejected. We performed the aforementioned permutation test on all the compounds examined. Examining the accounting for multiple comparisons (32 compounds) by Bonferroni correction, we set the significance level at 0.05/32 = 0.0015. At this level of significance, we found that the test was significant for all compounds.

**Figure 3:**
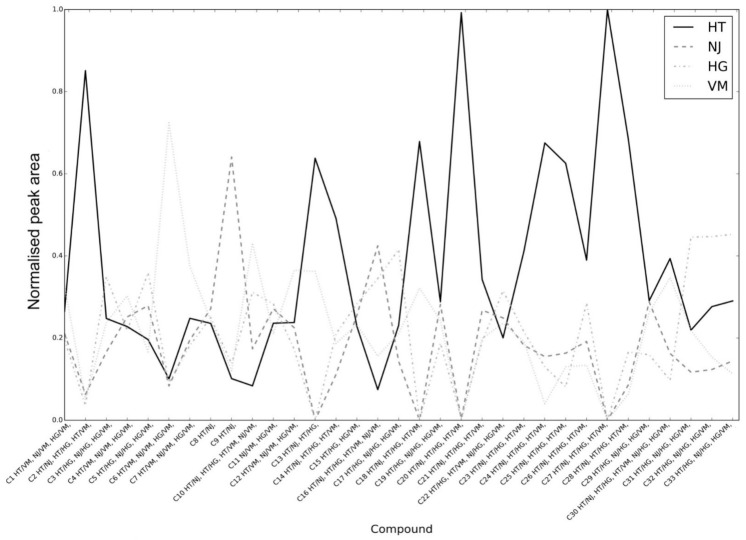
Plot of the mean of the peak area of each variety along the different compounds (with the magnitude normalized by the sum of all varieties) vs. the results of the significant pairwise tests for each compound. Abbreviations: HT, HT-801; HG, hybrid green F1; NJ, NJ-953; VM, “verde morado”.

**Figure 4:**
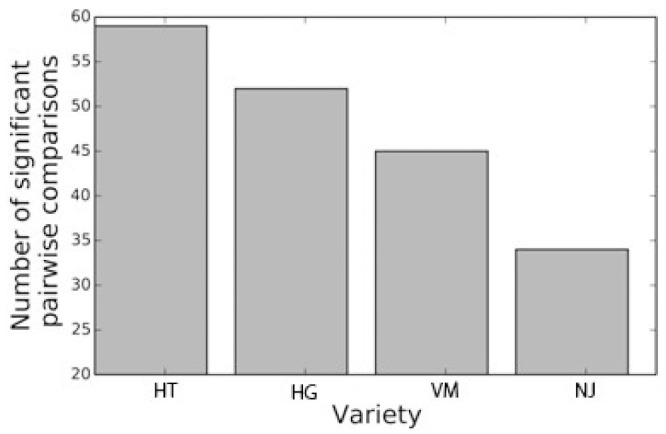
Permutation testing example for compound **1**. The empirical distribution of the F-statistic established by permutations for this compound can be seen in black. The value of the observed statistic is shown in gray. Abbreviations: HT, HT-801; HG, hybrid green F1; NJ, NJ-953; VM, “verde morado”.

Then, we examined the pairwise differences within compounds. For each compound and each pairwise combination of the varieties, we performed an F-test test for which the null hypothesis states that the mean of the two varieties is equal. This can be seen as the aforementioned permuted ANOVA test restricted to two varieties (instead of four). Since the F-test defaults to a t-test for the case of two groups, it can also be seen as a permuted t-test. In this case, the significance level of 0.05 was Bonferroni-corrected for the number of comparisons (188) and set to 0.05 / 188 = 0.0002. The result of this test can be seen in Figure 3 and Figure 4. Figure 3 displays the mean of each variety for the different compounds, with the magnitude normalized by the sum of all the varieties. For each compound, the results of the pairwise tests are presented on the x-axis. For any pair of varieties X and Y, X/Y in the x-axis denotes that the test has a *p*-value below the significance level. For example, HT/VM in compound **1** (l-glutamine) denotes that the mean of HT significantly differs from the mean of VM in that compound. It is striking that some compounds seem to be present in significantly higher amounts in a specific variety than in the others. For example, compounds **2** (l-asparagine), **13** (feruloylhexosylhexose isomer 1), **14** (feruloylquinic acid), **18** (feruloylhexosylhexose isomer 2), **20** (quercetin rhamnosyl rutinoside isomer 1), **24** (quercetin rhamnosyl rutinoside isomer 2), **25** (isorhamnetin glucosyl rutinoside), **26** (kaempferol-3-*O*-rutinoside (nicotiflorin)), **27** (isorhamnetin-3-*O*-glucoside), and **28** (isorhamnetin-3-*O*-rutinoside (narcissin)) are present in significantly higher amounts in HT than in the other three varieties. In addition, compounds **1** (l-glutamine), **4** (l-glutamic acid), **6** (xylonic/arabinonic/ribonic acid), **7** (hexosyl hexose), and **12** (*p*-coumaroylquinic acid) are present in significantly higher amounts in VM than in the other three varieties. Furthermore, compounds **13** and **18** (feruloylhexosylhexose isomers 1 and 2 respectively) were detected only in “triguero” varieties HT and VM, and compound **27** (isorhamnetin-3-*O*-glucoside) only in HT but not in any of the commercial hybrid green varieties. Therefore, these compounds are proposed as possible varietal markers. It is remarkable that compounds consisting of ferulic and/or coumaric acid esterified to a glycerol backbone (compounds **31**–**33**) are present in significantly higher amounts in HG than in the other three varieties.

In our study, we determined that the two “triguero” varieties (the improved HT followed by its parent “verde-morado”) are more significantly different than the other two commercial hybrid green varieties. This suggests that these landraces possess a richer phytochemical content than hybrid green ones. This is in line with other studies that have shown that the contents and the types of phenolic compounds, fatty acids, and saponins [4,32,33] are higher in “triguero” varieties than in other commercial green ones.

Furthermore, numerous scientific publications have provided evidence that indicates asparagus crude extracts may have antitumor [34], hypocholesterolemic and hepatoprotective [6,35], hypotensive and renal protective [36], and antidiabetic [37] effects. Furthermore, hypolipidemic effects have also been reported for fiber and flavonoid fractions in rats under a high-fat diet [6]. Discriminating consumers are increasingly demanding health-promoting food products. Therefore, new asparagus varieties with higher phytochemical content could be of great interest for the revalorization of this product.

## 3. Materials and Methods

### 3.1. Chemicals and Apparatus

All chemicals were of analytical reagent grade and used as received. Methanol was purchased from Panreac (Barcelona, Spain) and acetic acid from Sigma-Aldrich (Steinheim, Germany). Double-deionized water with conductivity lower than 18.2 MΩ was obtained with a Milli-Q system (Millipore, Bedford, MA, USA). The evaporation to dryness of the extracts was performed using a rotary vacuum evaporator model R-200 coupled to a heating bath model B-490, both from Büchi Labortechnik (Flawil, Switzerland.)

### 3.2. Samples

Asparagus spears harvested in Huétor Tájar region of Granada (Spain) were provided by Centro Sur S.A. (Cesurca). For this study, four varieties were used:

Commercial hybrid varieties: hybrid green F1 (HG), and NJ-953 (NJ).
“Triguero” varieties: “verde-morado” (VM), and HT-801 (HT).

They were harvested at ripeness and collected randomly from the field in 5-kg batches each to guarantee the representativity of the sample. Asparagus spears were cleaned with tap water, freeze-dried in a lyophilizer (Christ Alpha 1–2 LD Freeze dryer, Shropshire, UK), and kept at −18 C until use.

### 3.3. Extract Preparation

Of each freeze-dried sample, 0.5 g were dissolved in 15 mL of 80:20 (*v/v*) methanol/water and sonicated for 30 min at room temperature. A temperature control was carried out at 37–40 °C maximum to avoid any degradation of metabolites. Then, the mixture was centrifuged for 15 min at 3500 rpm and the supernatant was collected in a round-bottomed flask. The final volume was dried in a rotary evaporator under reduced pressure at 35 °C and the residue was reconstituted in 2 mL of 80:20 (*v/v*) methanol/water. Finally, the extract was filtered through a 0.2-µm PTFE syringe filter and kept at -20 °C prior to analysis. Three replicates were prepared from each sample.

### 3.4. Analytical Characterization

The asparagus extracts were analytically characterized by high-performance liquid chromatography coupled to electro-spray time-of-flight mass spectrometry (HPLC-DAD-ESI-TOF/MS). The HPLC-DAD-ESI-TOF/MS method was performed in an Agilent 1200-HPLC system (Agilent Technologies, Waldbronn, Germany) of the Series Rapid Resolution equipped with a vacuum degasser, autosampler, a binary pump, and a diode-array detector (DAD). The chromatographic separation was performed in a Zorbax Eclipse Plus RP-C18 analytical column (Agilent Technologies, Palo Alto, CA, USA) 150 × 4.6 mm i.d., 1.8 μm particle size). The flow rate was 0.80 mL/min, and the temperature of the column was maintained at 25 °C. The mobile phase used was water with 0.25% acetic acid as eluent A, and methanol as eluent B. The total run time was 27 min using the following multistep linear gradient: 0 min, 5%B; 7 min, 35%B; 12 min, 45%B; 17 min, 50%B; 22 min, 60%B; 25 min, 95%B, 27 min, 5%B, and finally a conditioning cycle of 5 min with the same conditions for the next analysis [38]. The injection volume in the HPLC was 10 μL, and each sample was injected in triplicate. The compounds separated were monitored in sequence first with DAD (240, 280, and 350 nm) and then with a mass-spectrometry detector. The MS analysis was performed using the microTOF (Bruker Daltonik, Bremen, Germany), which was coupled to the HPLC system. The TOF mass spectrometer was equipped with an ESI interface (model G1607A from Agilent Technologies, Palo Alto, CA, USA) operating in negative ion mode. External mass-spectrometer calibration was performed with sodium acetate clusters (5 mM sodium hydroxide in water/2-propanol 1/1 (*v/v*), with 0.2% of acetic) in quadratic high-precision calibration (HPC) regression mode. The optimum values of source and transfer parameters were established according to [38].

### 3.5. Permutation Test

Permutation testing was done in the Python programming language using routines from the libraries NumPy v1.9 [39] and SciPy v0.16 [40]. The NumPy libraries were used for fast array computing while SciPy was used for its statistical functions (F test) in the permutation tests. In total 100,000 permutations were conducted in this test, assuring a control for a significance level of 0.05. Figure 2, Figure 3 and Figure 4 were also produced within the Python language using the Matplotlib v1.4 plotting library [41].

## 4. Conclusions

Focusing on the phytochemical profile of the varieties in study (HT, HT-801; HG, hybrid green F1; NJ, NJ-953; VM, “verde morado”), we postulate that “triguero” varieties (the improved HT followed by its parent “verde-morado”) had a distinct phytochemical profile compared to the green commercial hybrid varieties (HG, NJ). Although more studies relating the phytochemical content of “triguero” asparagus varieties with its health-promoting effects are required, this characteristic phytochemical profile can be used for differentiating and revalorizating these asparagus cultivars.

## Data Availability

All the data generated by this research have been included in the article. It is possible to contact the corresponding author for any request of information.

## Supplementary Materials

The following are available online, Table S1: HPLC-ESI-TOF/MS data on the compounds identified. From left to right: compound number, retention time, experimental *m/z*, molecular formula, calculated *m/z*, error (ppm), maximum wavelength, proposed compound, and peak area for each variety title.

## Sample Availability

Not available.
