# Supplementary material for: Identification of Bioactive Compounds of Asparagus officinalis L.: Permutation Test Allows Differentiation among “Triguero” and Hybrid Green Varieties
Source: Molecules. 2021 Mar 15;26(6):1640. doi: 10.3390/molecules26061640 (PMC7999721; doi:10.3390/molecules26061640)
Supplement: Supplementary file 1 [file molecules-26-01640-s001.zip › molecules-1121219-supplementary.pdf]

C	RT (min)	m/z exp. (M-H)	Molecular formula (M-H)	m/z calc. (M-H)	Error (ppm)	λ	Proposed compound	Peak area			
								HT	NJ	HG	VM
1	1.976	145.0621	$C_5H_{10}N_2O_3$	145.0619	-1.3	210, 265	L-glutamine	2292260±19040	1825419±155114	1698453±140672	2795044±133643
2	2.063	131.0471	$C_4H_7N_2O_3$	131.0462	-6.9	212, 265	L-asparagine	8506428±469622	627495±7625	361454±7870	489984±24187
3	2.077	135.0295	$C_4H_8O_5$	135.0299	2.6	n.d.	Threonic acid	542394±27208	364250±14874	763587±8434	518670±6867
4	2.077	146.0472	$C_5H_9NO_4$	146.0459	-8.9	212, 265	L-glutamic acid	1322409±34459	1458807±92003	1265847±154273	1724694±97441
5	2.127	191.0565	$C_7H_{12}O_6$	191.0561	-1.8	n.d.	Quinic acid	1487200±111596	2103974±142135	2719505±178366	1278566±90260
6	2.145	165.0407	$C_5H_{10}O_6$	165.0405	1.2	212, 265	Xylonic/arabinonic/ribonic acid	844451±13701	669761±18551	766715±8147	6096329±54176
7	2.176	341.1092	$C_{12}H_{22}O_{11}$	341.1089	-0.7	214, 267	Hexosyl hexose	4147403±263297	3267514±98770	3043562±105108	6270930±79304
8	2.244	209.0671	$C_7H_{14}O_7$	209.0667	-1.9	n.d.	Sedoheptulose (altro-heptulose)	1832113±75996	2107331±106624	1942958±94105	1883125±53373
9	2.427	133.0138	$C_4H_6O_5$	133.0142	3.5	n.d.	Malic acid	529898±65921	3349509±92641	718547±6176	628267±12869
10	8.079	353.0899	$C_{16}H_{18}O_9$	353.0878	-6.0	221, 236, 296, 323	Caffeoylquinic acid (chlorogenic acid)	2736023±75731	5690792±45203	10169161±120618	14541919±1563909
11	9.350	311.0287	$C_{13}H_{12}O_9$	311.0409	38.9	238, 296, 324	Caffeoyltartaric acid (caftaric acid) isomer 1	1132550±28334	1313382±77596	1357182±112509	1020386±81591
12	9.351	337.0939	$C_{16}H_{18}O_8$	337.0929	-2.2	235, 314	p-coumaroylquinic acid	2793126±44355	2659591±252746	2025679±104521	4270106±269607
13	9.501	517.1565	$C_{22}H_{30}O_{14}$	517.1565	-0.0	n.d.	Feruloylhexosylhexose isomer 1	871332±81449	0±0	0±0	500847±26047
14	9.884	367.1060	$C_{17}H_{20}O_9$	367.1035	-6.8	234, 296, 324	Feruloylquinic acid	23635588±730469	5366567±220900	10131041±503659	9026796±355690
15	10.153	311.0278	$C_{13}H_{12}O_9$	311.0409	42.1	238, 296, 324	Caffeoyltartaric acid (caftaric acid) isomer 2	1267822±23486	1407048±82355	1573148±122746	1341883±191169
16	10.304	355.1041	$C_{16}H_{20}O_9$	355.1035	-1.8	n.d.	Feruloylhexose	699853±19067	3979146±35012	3235044±64694	1453456±93822

17	10.324	473.0828	$C_{22}H_{18}O_{12}$	473.0725	-21.6	n.d.	Dicaffeoyltartaric acid (chicoric acid)	923721±48233	567702±30051	1661990±83128	834010±4073
18	10.353	517.1582	$C_{22}H_{30}O_{14}$	517.1565	-3.6	n.d.	Feruloylhexosylhexose isomer 2	745087±16083	0±0	0±0	349217±10391
19	12.327	625.1417	$C_{27}H_{30}O_{17}$	625.1410	-1.1	248, 266	Quercetin diglucoside	9507402±52389	9282472±85499	6190374±639911	7994585±101437
20	13.363	755.2073	$C_{33}H_{40}O_{20}$	755.2040	-4.4	n.d.	Quercetin rhamnosyl rutinoside isomer 1	20127599±663042	31526±2106	45637±10300	0±0
21	14.918	771.1985	$C_{33}H_{40}O_{21}$	771.1989	0.5	255, 265, 355	Quercetin glucosyl rutinoside	7624301±160923	5921412±297805	4266983±130022	4307962±387431
22	16.038	609.1481	$C_{27}H_{30}O_{16}$	609.1461	-3.2	255, 267, 355	Quercetin-3-O-rutinoside (rutin)	54082180±1154936	67130326±657784	84873352±1162535	63630856±1000844
23	16.122	463.0914	$C_{21}H_{20}O_{12}$	463.0882	-6.8	255, 355	Quercetin-3-O-glucoside	1363837±42235	601934±42307	713604±70798	626092±37605
24	16.791	755.2039	$C_{33}H_{40}O_{20}$	755.2040	0.2	263, 355	Quercetin rhamnosyl rutinoside isomer 2	401100±7077	90144±6999	77121±4413	24345±2226
25	17.227	785.2147	$C_{34}H_{42}O_{21}$	785.2146	-0.1	560, 353	Isorhamnetin glucosyl rutinoside	637690±2404	166094±5710	83053±7517	129876±15393
26	18.397	593.1529	$C_{27}H_{30}O_{15}$	593.1512	-2.9	265, 355	Kaempferol-3-O-rutinoside (nicotiflorin)	6758127±458067	3292686±204097	4941639±161068	2257003±232401
27	18.597	477.1054	$C_{22}H_{22}O_{12}$	447.1038	-1.6	255, 265, 353	Isorhamnetin-3-O-glucoside	703228±45383	0±0	0±0	0±0
28	18.882	623.1652	$C_{28}H_{32}O_{16}$	623.1618	-5.5	255, 265, 355	Isorhamnetin-3-O-rutinoside (narcissin)	13512891±638437	1673614±172307	3278567±155844	1218339±157905
29	19.432	263.1290	$C_{15}H_{20}O_{4}$	263.1289	-0.4	262	Abscisic acid	205291±5496	202334±3110	112499±12000	183142±9516
30	22.361	421.2115	$C_{19}H_{34}O_{10}$	421.2079	-8.6	265, 368	Unknown	834503±8560	343858±12778	206589±9505	730735±19417
31	25.001	383.1164	$C_{21}H_{20}O_{7}$	383.1136	-7.2	246, 300, 317	Dicoumaroyl glycerol	1141389±39071	607830±14666	2319478±76753	1161059±139362
32	25.019	413.1272	$C_{22}H_{22}O_{8}$	413.1242	-7.2	251, 296, 315	Coumaroyl feruloyl glycerol	2338362 ±34428	1040980±27051	3780796±172626	1293633±57152
33	25.034	443.1368	$C_{23}H_{24}O_{9}$	443.1348	-4.6	251, 300, 313	Diferuoloyl glycerol	437032±5577	216524±6863	681266±30491	165814±11784

Table S1. HPLC-ESI-TOF/MS data on the compounds identified. From left to right: compound number, retention time, experimental *m/z*, molecular formula, calculated m/z, error (ppm), maximum wavelength, proposed compound, and peak area for each variety.
